# Changes in Intrinsic Activity of the Primary Somatosensory Cortex Causally Explain Differences in Emotion Perception in Autism

**DOI:** 10.1002/aur.70197

**Published:** 2026-02-13

**Authors:** Martina Fanghella, Danai Dima, Dimitrios Pinotsis, Sebastian B. Gaigg, Beatriz Calvo‐Merino, Bettina Forster

**Affiliations:** ^1^ Psychology & Neuroscience Department, School of Health and Medical Sciences City St George's, University of London London UK; ^2^ Cognition in Action (CIA) Unit, PHILAB, Department of Philosophy University of Milan Milan Italy; ^3^ Department of Neuroimaging Institute of Psychiatry, Psychology and Neuroscience, King's College London London UK

**Keywords:** autism spectrum disorder, dynamic causal modeling, EEG, embodiment, emotion, somatosensory

## Abstract

Autism Spectrum Disorder (ASD) is characterized by certain difficulties in emotion‐related processing. Recent research using electroencephalography (EEG) to measure somatosensory evoked potentials during emotion perception has shown reduced embodiment of emotional expressions in autistic compared to neurotypical individuals, independently from differences in visual processing. However, the underlying neural dynamics are not clear. In this study, we use Dynamic Causal Modeling (DCM) on EEG data to investigate whether reduced embodiment during emotion processing in ASD individuals is caused by changes in intrinsic connectivity within the somatosensory cortex, or by top‐down modulatory effects from higher‐order frontal areas. We constructed a model involving the primary and secondary right somatosensory cortex, the right supplementary motor area and the right inferior frontal gyrus, and tested effective connectivity during emotion or gender discrimination tasks in two groups of ASD and typically developing (TD) participants (*n* = 38, male and female, 2 females). Our results reveal that task‐related differences in electrocortical activity between the emotion and gender tasks are causally explained by changes in intrinsic activity within the right primary somatosensory cortex (rS1) in both TD and ASD. Importantly, these intrinsic changes in rS1 are significantly different between TD and ASD groups and individual task‐related changes in rS1 significantly correlate with alexithymia traits. Our study provides novel evidence on the neural dynamics underlying difficulties in emotion processing in ASD individuals, highlighting that differential intrinsic activations of the rS1 are causally involved in such difficulties, and suggests that they are mediated by alexithymia.

## Lay Summary

Autism is often characterized by difficulties in perceiving and recognizing emotions. These differences involve not only visual perception, but also somatic, visceral, and motoric re‐enactment of the observed emotion (embodiment). Our results highlight that differences in emotion embodiment in autism are explained by changes in the right primary somatosensory cortex, and they are mediated by alexithymia.

## 1 Introduction

Understanding others' emotional expressions is a fundamental aspect of successful social interactions. Perceiving others' emotions involves perceptual, somatovisceral, and motoric representation of the emotion in one's self, defined as embodiment (Niedenthal 2007). It is implemented in a distributed network of cortical and subcortical areas, including sensory‐motor areas (Pessoa and Adolphs 2010; Schirmer and Adolphs 2017; Underwood et al. 2021).

### 1.1 Somatosensory Processing of Emotions

The role of the somatosensory cortex in emotion embodiment has been supported by fMRI studies (Carr et al. 2003; Hennenlotter et al. 2005; Kragel and LaBar 2016; van der Gaag et al. 2007; Volynets et al. 2020) and neuromodulation studies. Specifically, it has been shown that applying TMS to the right primary somatosensory cortex (rS1) during an emotion discrimination task disrupts emotion recognition (Pitcher et al. 2008; Pourtois et al. 2004). Similarly, research on patients has demonstrated reduced emotion discrimination in individuals with right parietal lesions including somatosensory areas (Adolphs et al. 1996, 2000; Atkinson and Adolphs 2011). Finally, independent contributions of the somatosensory cortex to emotion processing have been shown by recent ERP studies (Arslanova et al. 2023; Sel et al. 2014, 2020), which combined visual and somatosensory evoked potentials to dissociate somatosensory activity during emotion perception from visual carryover effects (Galvez‐Pol et al. 2020).

### 1.2 Embodiment of Emotions in ASD

Autism Spectrum Disorder (ASD) is characterized by difficulties in emotion‐related processing (see (Gaigg 2012) for a review). Importantly, a recent ERP study (Fanghella et al. 2022) revealed different somatosensory embodiment of emotional expressions in individuals with autism spectrum disorder (ASD) compared to typically developing (TD) controls. In this study, individuals with ASD exhibited significantly reduced Somatosensory Evoked Potential (SEP) P100 components during emotion recognition but not in a control gender task compared to a group of TD participants. Moreover, the strength of autistic traits correlated with the amplitudes of SEP P100 components in the emotion, but not in the gender task, in ASD individuals and in the whole sample.

Two recent studies investigating embodied emotion perception in TD individuals showed modulation of beta desynchronization localized to somatosensory areas by anxiety and autistic traits (Charidza and Gillmeister 2022) and modulation of somatosensory processing of emotional expressions by levels of alexithymia (Arslanova et al. 2023). Together, these studies highlight that somatosensory embodiment of emotions can be modulated by personality traits and may operate differently in neurodevelopmental or psychiatric conditions.

Although there is consistent evidence for diminished embodiment of others' emotions (Fanghella et al. 2022; Masson et al. 2019), social touch (Lee Masson 2025; Masson et al. 2019) or pain (Minio‐Paluello et al. 2009) in ASD, the neural dynamics underlying such differences are still not fully understood.

Indeed, it is still not clear if decreased emotion embodiment in ASD is associated with reduced activations within the somatosensory cortex, or if these differences are driven by atypical top‐down modulations between high‐order frontal areas and the somatosensory cortex. In line with this hypothesis, a recent fMRI study (Isakoglou et al. 2023) assessed the connectivity of the primary somatosensory cortex (S1) and other brain areas during resting state and while performing an emotional matching task in two groups of ASD and TD individuals. Results suggested that S1 activations during emotion processing were influenced in a top‐down manner, and changes in connectivity were mediated by ASD traits.

### 1.3 Dynamic Causal Modeling of EEG Data

In this study, we used Dynamic Causal Modeling (DCM) (Friston et al. 2003) to elucidate the dynamics underlying differences in EEG responses during emotion perception in two groups of ASD and TD participants. DCM is a biologically plausible model which can explain empirical ERP phenomena in terms of changes in connectivity among distinct cortical sources (David et al. 2006). Changes in source connections may be intrinsic (within source) and extrinsic (between sources). Intrinsic connections model adaptation of neuronal responses to local influences, while forward and backward connections are mediated by long‐range (respectively bottom‐up or top‐down) between‐area extrinsic connections (Kiebel et al. 2007).

Here, DCM was employed to investigate whether reduced somatosensory activations during emotion perception in ASD are caused by changes in intrinsic connectivity of the somatosensory cortex or are a by‐product of modulatory effects from other brain regions involved in embodiment of emotions, in particular top‐down modulations from high‐order frontal areas (i.e., supplementary motor area and inferior frontal gyrus), as other studies (Isakoglou et al. 2023) have suggested.

Importantly, DCM for ERP has been used to model differences in electrophysiological responses between clinical populations and healthy controls, in particular schizophrenia (Braeutigam et al. 2018; Dima et al. 2010; Fogelson et al. 2014; Hüpen et al. 2025; Ranlund et al. 2016). DCM has also been used to model fMRI BOLD activity from ASD and control participants (Bird et al. 2006; Cook et al. 2012), in particular during facial emotion perception (Sato et al. 2019) and affective body gestures (Grèzes et al. 2009). Yet, no previous DCM studies have investigated alterations in sensorimotor responses during emotion perception in individuals with ASD. Moreover, to our knowledge, no studies have been conducted using DCM for EEG to model differences between ASD and TD participants.

### 1.4 Dynamic Causal Modeling of Emotion Perception in ASD

In our study, we re‐analyzed with DCM an EEG dataset from ASD and TD individuals while performing a facial emotion discrimination task and a control gender task (Fanghella et al. 2022). After isolating somatosensory responses from visual carryover effects (Sel et al. 2014), we used DCM to investigate whether reduced somatosensory responses in ASD during emotion embodiment are caused by changes within the somatosensory cortex, or if these differences are driven by modulatory effects from higher‐order brain areas. In fact, simple ERP analysis cannot make inferences on the cortical neural network underlying somatosensory responses. Importantly, we modeled somatosensory responses free from visual carryover effects (VEP‐free SEPs) to probe the dynamics of emotion embodiment beyond visual processing of emotional expressions (Fanghella et al. 2022) using DCM.

To answer this question, we constructed a model lateralized on the right hemisphere (Adolphs et al. 1996, 2000; Pitcher et al. 2008), involving the primary (rS1) and secondary (rS2) right somatosensory cortex, the right supplementary motor area (rSMA) and the right inferior frontal gyrus (rIFG), and we tested effective connectivity during emotion processing in two groups of ASD and TD participants. Specifically, we selected these areas based on previous source reconstruction of this EEG dataset, showing distributed cortical sources in somatosensory and high‐order motor areas (Fanghella et al. 2022) and on previous literature describing the involvement of sensorimotor areas and the IFG in mirroring emotional expressions (Bastiaansen et al. 2009; Carr et al. 2003; Dapretto et al. 2006; van der Gaag et al. 2007). We restricted the model to the right hemisphere consistently with evidence from TMS (Pitcher et al. 2008; Pourtois et al. 2004) and lesion studies (Adolphs et al. 1996, 2000). Then, we added extrinsic (forward and backward) and intrinsic connections to the model to explore any possible modulation between and within areas.

We did not include cortical areas (e.g., the fusiform face area, superior temporal sulcus) involved in emotion processing because they are not implicated in emotion embodiment (Ganel et al. 2005; Meaux et al. 2014; Pourtois et al. 2004; Sato et al. 2015; Vuilleumier and Pourtois 2007).

## 2 Methods

### 2.1 Participants

Forty‐four adult participants, half with a diagnosis of ASD and the other half TD adults matched for IQ, age, and gender, took part in the experiment. Previous research confirmed that this is an adequate sample size for DCM studies (Goulden et al. 2012; Kasess et al. 2010; Ma et al. 2024), and it is commonly used to model differences between clinical populations and controls (Braeutigam et al. 2018; Dima et al. 2010; Hüpen et al. 2025; Sato et al. 2019). The Local Ethics Committee approved all research methods, which were carried out following the principles of the revised Helsinki Declaration (World Medical Association 2013). Written informed consent was obtained from all the participants. All participants had normal or corrected‐to‐normal vision. We administered all participants with a short version of the Wechsler Adult Intelligence Scale and obtained a Verbal IQ (VIQ) and Performance IQ (PIQ) for each participant and ensured no significant group differences in VIQ and PIQ. All participants completed the adult self‐report form of the Social Responsiveness Scale, second edition (SRS‐2) (Constantino and Gruber 2012) and the Autism Quotient (AQ) (Baron‐Cohen et al. 2001), assessing the strength of autistic traits, the 20‐items Toronto Alexithymia Scale (TAS‐20) (Taylor et al. 2003), quantifying alexithymia traits, and the Multidimensional Assessment for Interoceptive Awareness, second edition (MAIA‐2) (Mehling et al. 2018), measuring interoceptive awareness. For a summary of demographics and questionnaires' scores, see Table S1. Datasets from two participants (1 ASD, 1 TD) were not included in the final analysis because stimulus markers were not recorded in the EEG recording during data collection. We excluded two additional ASD participants because of excessive artifacts in their EEG data (drift because of sweat and artifacts caused by muscular tension) and two TD participants because they scored above cut‐off on the Social Responsiveness Scale (SRS‐2, cut‐off above 60 (Bölte et al. 2011)) and Autism Quotient (AQ, cut‐off above 32 (Baron‐Cohen et al. 2001)), respectively. The final sample was thus composed of 19 ASD (17 right‐handed, 1 female) and 19 TD participants (19 right‐handed, 1 female).

### 2.2 Stimuli

We used a set of pictures depicting neutral, fearful, and happy emotions used in a previous study (Sel et al. 2014), originally selected from the Karolinska Directed Emotional Faces set (Lundqvist et al. 1998). The gray‐scaled faces were enclosed in a rectangular frame (140 × 157 in.), excluding most of the hair and non‐facial contours.

### 2.3 Task

**FIGURE 1:**
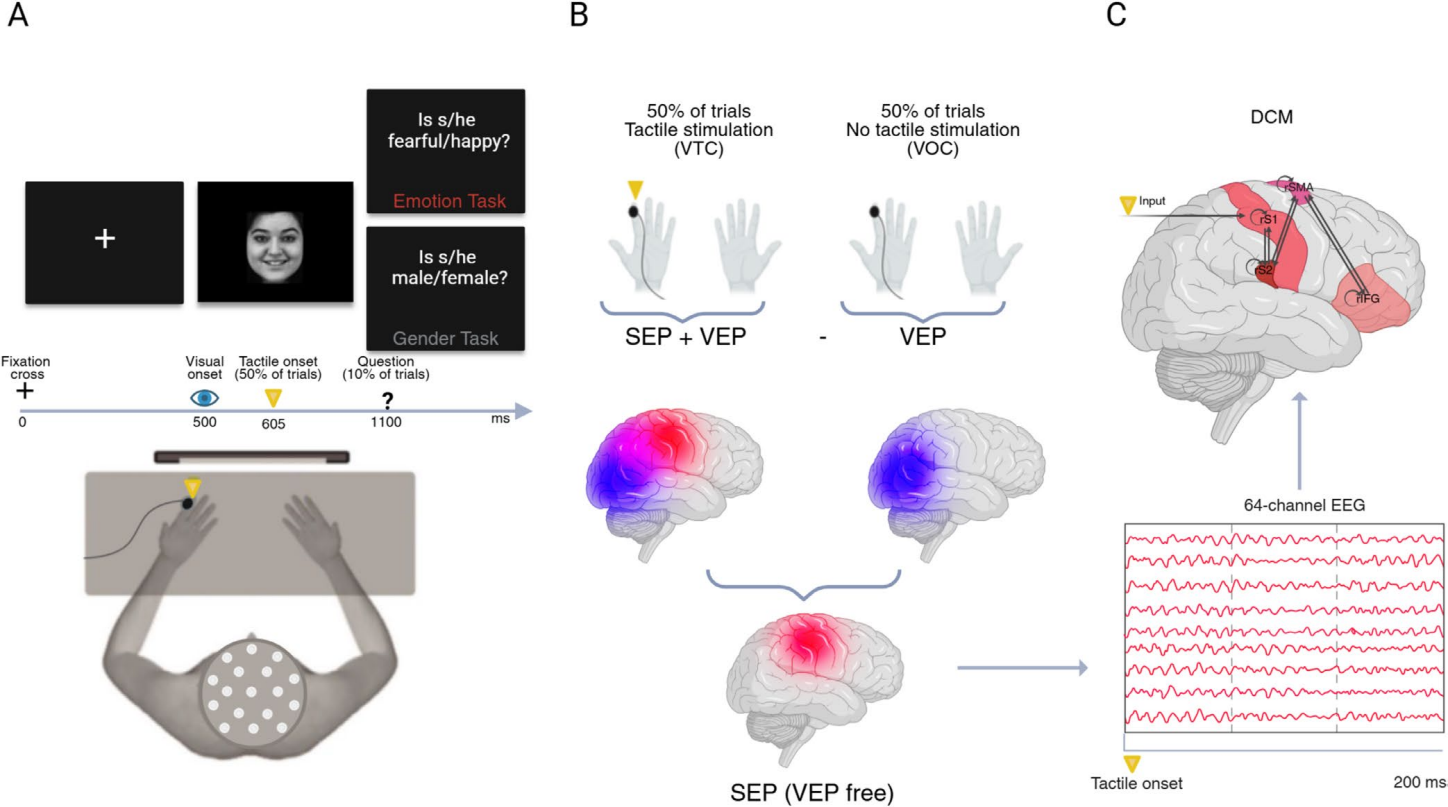
(A) Experimental task. Participants observed faces expressing neutral, fearful of happy emotions, and focused on the emotional expression (emotion task) or the gender (gender task). In 50% of trials, a task‐irrelevant tactile stimulation was delivered on the left index finger 105 ms after visual onset. (B) Subtraction of visual evoked potentials (VEP, 50% of trials) from visual + somatosensory evoked potentials (VEP + SEP; 50% of trials) to isolate somatosensory activity during emotion processing from concurrent visual carryover effects (VEP‐free SEP). (C) Dynamic causal modeling (DCM) of VEP‐free SEP. We hypothesized a hierarchical model involving primary (rS1) and secondary (rS2) right somatosensory cortex, right supplementary motor area (rSMA) and right inferior frontal gyrus (rIFG), and we performed DCM on 64‐channel EEG (*VEP‐free SEP)* on a 200‐ms time window after tactile onset. Created in BioRender. Fanghella, M. (2025) https://BioRender.com/y03g197.

Participants sat in an electrically and acoustically shielded chamber (Faraday's cage) in front of a monitor at a distance of 80 cm. Visual stimuli were presented centrally on a black background using E‐Prime software (Psychology Software Tools). Trials started with a fixation cross (500 ms), followed by the presentation of a face image (neutral, fearful, or happy, either male or female) for 600 ms. The experiment consisted of 1200 randomized trials, presented in two separate blocks of 600 trials, which included 200 neutral, 200 fearful, and 200 happy faces (half male and half female), presented in randomized order. In the emotion task (block 1), participants were instructed to attend to the emotional expression of the faces, while in the gender task (block 2) they needed to attend to the gender of the faces. The order of presentation of the two blocks was counterbalanced across participants. To ensure participants were attending to the stimuli, in 10% of emotion block trials, participants were asked whether the face stimulus was fearful (Is s/he fearful?) or happy (Is s/he happy?), or whether it depicted a female (Is s/he female?) or male (Is s/he male?) during the gender block trials. When a question was presented, participants had to respond vocally (yes/no) as soon as possible. Responses were recorded with a digital recorder and manually inserted by the experimenter, who was able to hear the participant from outside the Faraday's cage through an intercom. Before starting each block, participants completed a practice session with 12 trials (four neutral, four happy, four fearful, half male and half female). To probe somatosensory activity and evoke SEP during the task, in 50% of trials (Visual‐Tactile Condition, VTC), participants received task‐irrelevant tactile taps on their left index finger 105 ms after face images onset (Sel et al. 2014). In the Visual‐Only Condition (VOC, 50% of trials), the same visual facial stimuli were presented without any concurrent tactile stimulation (for a graphical illustration of the task, see Figure 1A). VTC and VOC were equally distributed in each block across the stimulus types (emotion, gender). Tactile taps were delivered using a 12 V solenoid driving a metal rod with a blunt conical tip that contacted participants' skin when a current passed through the solenoid. Participants were instructed to ignore the tactile stimuli. To mask sounds made by the tactile stimulator, we provided white noise through one loudspeaker placed 90 cm away from the participants' head and 25 cm to the left side of the participants' midline (65 dB, measured from the participants' head location with respect to the speaker).

### 2.4 EEG Recording and Data Preprocessing

We recorded EEG from a 64‐electrode cap (M10 montage; EasyCap). All electrodes were online referenced to the right earlobe and offline re‐referenced to the average of all channels. Vertical and bipolar horizontal electrooculogram and heartbeats were also recorded. Continuous EEG was recorded using a BrainAmp amplifier (BrainProducts; 500 Hz sampling rate). Analysis of the EEG data was performed using BrainVision Analyzer 2.2 software (BrainProducts). The data were digitally low‐pass‐filtered at 30 Hz and high‐pass‐filtered at 0.1 Hz. Ocular correction was performed (Gratton et al. 1983), and the EEG signal was epoched into 700 ms segments, starting 100 ms before tactile stimulus onsets. We performed baseline correction using the first 100 ms before tactile onset. Artifact rejection was computed eliminating epochs with amplitudes exceeding ±100 mV. We ensured that the signal‐to‐noise ratio was similar in the two groups (i.e., no significant difference in remaining number of epochs). Single‐subject grand‐averaged ERP for each task (emotion, gender) were computed. After preprocessing, single‐subject averages of VOC trials were subtracted from single‐subject averages of VTC trials, to isolate somatosensory evoked responses from visual carryover effects (Fanghella et al. 2022; Galvez‐Pol et al. 2020; Sel et al. 2014), see Figure 1B for a graphical depiction of this subtractive method.

### 2.5 Accuracy in Emotion/Gender Recognition Task

We extracted the mean accuracy for each participant, expressed in a value in a range between 0 (0% of correct answers) and 1 (100% correct answers). Exclusion criteria were set to accuracy < 50%. We computed a 2 × 2 mixed repeated‐measures ANOVA with Group (TD, ASD) as a between factor and Task (Emotion, Gender) as a within factor. This analysis has been previously reported in (Fanghella et al. 2022).

### 2.6 Amplitudes of SEP

We computed mean amplitudes of SEP‐ in four consecutive time windows of 30 ms length starting from 40 ms up to 160 ms after tactile stimulus onset (occurring after 105 ms of visual stimulus onset). These time windows were centered on the P50 (40–70 ms), N80 (70–100 ms), P100 (100–130 ms), and N140 (130–160 ms) peaks. Analyses were restricted to 18 electrodes located over sensorimotor areas (corresponding to FC1/2, FC3/4, FC5/6, C1/2, C3/4, C5/6, Cp1/2, Cp3/4, CP5/6, of the 10/10 system) (Sel et al. 2014). We selected the time windows from the grand average of all conditions and participants (Luck and Gaspelin 2017). SEP mean amplitudes were analyzed through mixed repeated‐measures ANOVAs in SPSS and JASP. Consistent with previous analyses (Sel et al. 2014), within‐group factors of the ANOVAs were as follows: Task (Emotion, Gender), Emotion (Neutral, Fearful, Happy), Hemisphere (Left, Right), Site (Dorsal, Dorsolateral, Lateral; i.e., clusters of three electrodes grouped in parallel to the midline), Region (Frontal, Central, Posterior; i.e., clusters of three electrodes grouped perpendicularly to the midline), and the between‐factor Group (TD, ASD). Follow‐up ANOVAs and two‐tailed independent and paired‐sample *t*‐tests were conducted to follow‐up significant interactions, and post hoc pairwise comparisons were computed on significant main effects. We applied Greenhouse–Geisser when appropriate (Keselman and Rogan 1980), and post hoc tests were corrected for multiple comparisons (Bonferroni). The full analysis is reported in (Fanghella et al. 2022).

### 2.7 Dynamic Causal Modeling

We performed Dynamic Causal Modeling (DCM) for EEG with SPM 12 (SPM12 Software—Statistical Parametric Mapping, n.d.) on VEP‐free SEP (Fanghella et al. 2022) to estimate connectivity among brain areas and how such connectivity is influenced by emotion or gender in a discrimination task in TD and ASD individuals (see Figure 1C). DCM explains EEG data using a hierarchical network of dynamically interacting sources, making predictions about the dynamics of each source and estimating effective connectivity using Bayesian model inversion (Friston et al. 2003).

### 2.8 DCMs Specification

**FIGURE 2:**
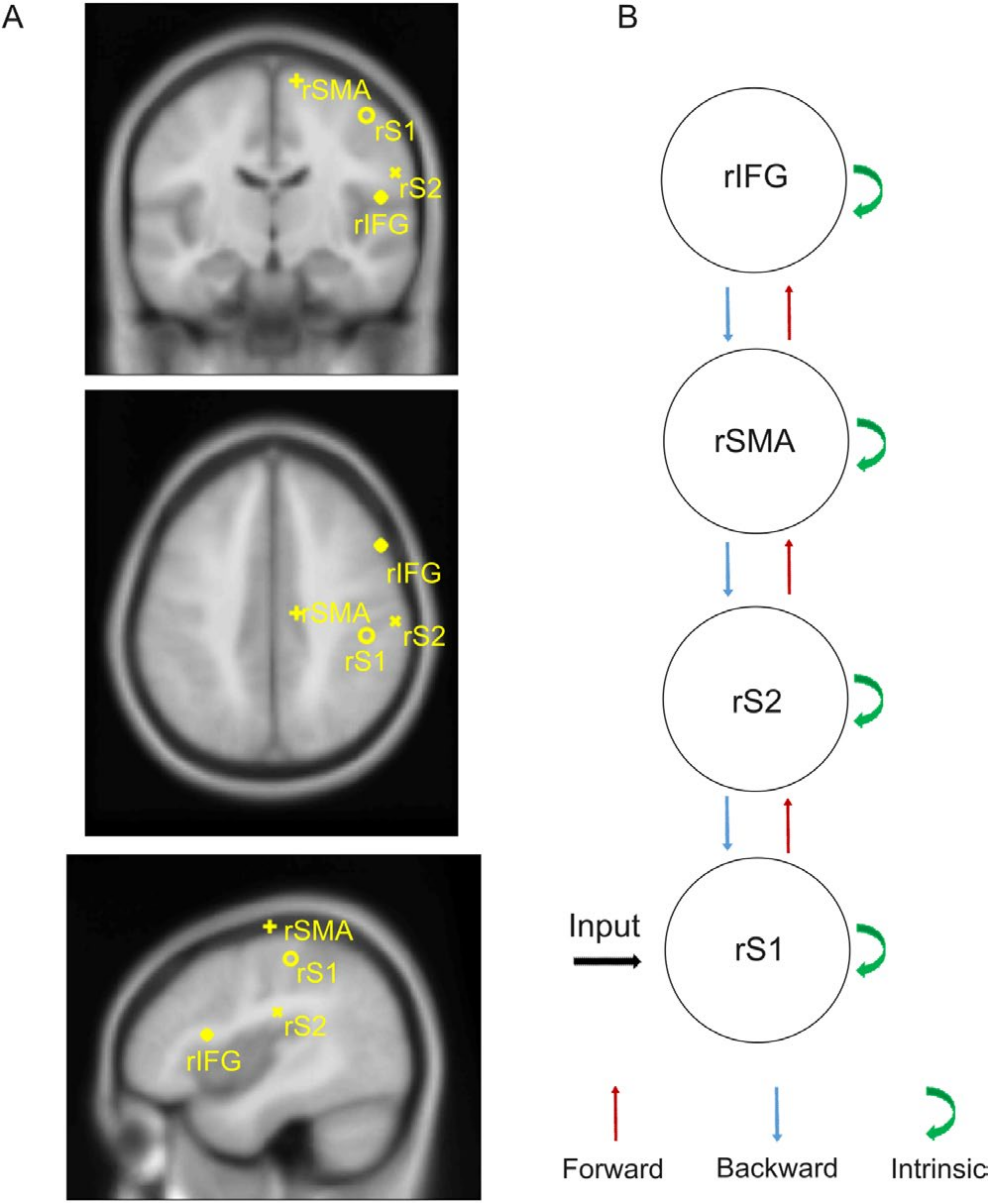
Model specification. The models have the same structural connectivity, but different modulation of effective connectivity according to the task (emotion or gender discrimination). (A) The sources included in the models were: rS1, right primary somatosensory cortex; rS2, right secondary somatosensory cortex; rSMA, right supplementary motor area; and rIFG, right inferior frontal gyrus. (B) The sources are linked by extrinsic (forward, red arrows and backward, blue arrows) connections, and each source has intrinsic modulations (green arrows). Task‐related changes in effective connectivity were tested across forward, backward and intrinsic modulations of all sources.

Our DCM model assumes the existence of extrinsic (forward and backward) connections between sources, and intrinsic connections within the specified sources (Pinotsis et al. 2019; Ranlund et al. 2016). The model consists of a four‐level lateralized hierarchy comprising the right primary somatosensory cortex (rS1), modeled as direct sensory input, the right secondary somatosensory cortex (rS2), the right supplementary motor area (rSMA), and the right inferior frontal gyrus (rIFG) (see Figure 2 for a graphical depiction of the model). Sensorimotor areas were selected among locations highlighted by source reconstruction of EEG data, performed on grand‐averaged ERP data for group (TD, ASD) and task (emotion, gender), as described in (Fanghella et al. 2022). The IFG was added to the model based on previous literature that looked at differences in emotion perception in TD and ASD individuals (Dapretto et al. 2006). Source coordinates in MNI are reported in Table 1. We hypothesized a hierarchical model with forward and backward connections between the areas of interest plus intrinsic modulations for each of the selected areas, and we tested all possible task‐related modulations on forward (bottom‐up), backward (top‐down), and intrinsic (within) connections.

**TABLE 1:** Sources MNI coordinates in XYZ.

	X	Y	Z
rS1	46	−29	54
rS2	60	−22	26
rSMA	12	−18	71
rIFG	53	15	14

Abbreviations: rIFG, right inferior frontal gyrus; rS1, right primary somatosensory cortex; rS2, right secondary somatosensory cortex; rSMA, right supplementary motor area.

The input of DCM models were individual grand‐averaged EEG data for each task (emotion and gender), including 200 ms epochs starting from tactile onset. SEP were previously isolated from visual carryover effects through subtractive method (Fanghella et al. 2022). This time window allowed us to capture the neural dynamics across all SEP components of interest (P50, N80, P100, N140). We selected the “SEP” connectivity model, a faster variant of the standard “ERP” model (Ashburner et al. 2014). We modeled each node of the model with a single equivalent current dipole (ECD). Input onset in the rS1 was set 20 ± 10 ms after tactile stimulus onset.

Coordinates of the rS1 and the rSMA (in MNI) were extracted from source reconstruction of the SEP components of interest (P50, N80, P100, and N140) (Fanghella et al. 2022), while coordinates of the rS2 and the rIFG were taken from previous studies using similar paradigms (rS2: (Conty et al. 2012); rIFG: (Carr et al. 2003), converted from Talairach to MNI system). We optimized source coordinates for each participant by selecting the option “optimize source locations,” allowing flexibility of prior location coordinates by relaxing zero variance priors on the specified source locations (DCM for Evoked Responses—SPM Documentation, n.d.).

### 2.9 DCM Model Comparison

We created three families of models. Each model had the same hierarchical structure but differed in task‐related modulations (depicted in Figure 2). To avoid any theory‐driven bias, our 10 models covered all possible connectivity configurations (forward‐intrinsic‐backward). The first family (*forward*) involved three models with task‐related changes in forward connectivity from rS1 to rS2, from rS2 to rSMA, and from rSMA to rIFG; the second family (*intrinsic*) included four models with task‐related intrinsic modulations of rS1, rS2, rSMA, and rIFG; the third family (*backward*) was composed of three models involving backward connections from rS2 to rS1, from rSMA to rS2, and from rIFG to rSMA. After inverting all models for each participant, we compared families and models using random‐effects (RFX) Bayesian Model Selection (BMS) (Stephan et al. 2009) for each group (ASD, TD) separately to select the best model among all possible configurations. In addition, to provide further evidence of the robustness of our results, we computed Bayesian Model Averaging (BMA) on the winning family (Penny et al. 2010). The best model was selected evaluating exceedance posterior probabilities (Stephan et al. 2009) and not protected posterior probabilities (Rigoux et al. 2014) because this method is not suitable for inference at the family level.

### 2.10 DCM Effective Connectivity Parameters

After inverting all DCM models and identifying the group‐level winning model through BMS, we extracted connectivity parameters (forward, intrinsic, and backward) from the winning model for each participant to compare how the task affected endogenous and effective connectivity in the two groups. Then, we computed the Shapiro–Wilk normality test and selected the appropriate statistical test (parametric or non‐parametric independent‐sample test) for each variable. For non‐parametric *t*‐tests, effect size is given by rank biserial correlation (Kerby 2014). Confidence Interval (CI) are reported.

We further explored the association between task‐related modulatory effects of the winning model and personality traits (autism, alexithymia and interoceptive awareness) by computing parametric or non‐parametric correlations between individual connectivity parameters and their scores in each personality questionnaire (SRS‐2, AQ, TAS‐20, and MAIA‐2). We computed this analysis first on the whole sample of participants, and then on the two groups separately. For each questionnaire, we Bonferroni‐corrected *p*‐values for multiple comparisons (0.05/3 = 0.017) and set 0.017 as the significance threshold. For non‐parametric correlations, effect size is given by Spearman rho (*ρ*). Confidence Interval (CI) from 1000 bootstraps is reported.

To ensure the robustness of results, we replicated all statistical analyses also on connectivity parameters extracted from BMA of the winning family.

All statistical analyses are computed with the software JASP 0.18.3 (JASP Team (2024). JASP (Version 0.19.3). n.d.).

## 3 Results

### 3.1 Accuracy in Emotion/Gender Recognition Task

The analysis revealed no task‐dependent differences in accuracy between the two groups.

The mixed repeated‐measures ANOVA showed a significant main effect of Group (F(1,36) = 5.396, *p* = 0.026, η_p_^2^ = 0.130), explained by an overall decreased accuracy for the ASD compared with the TD group. No further significant effects were found (main effect of Task, F(1,36) = 0.751, *p* = 0.392, η_p_^2^ = 0.020, Group × Task interaction, F(1,36) = 1.827, *p* = 0.185, η_p_^2^ = 0.048), suggesting that the behavioral differences between the two groups were not task dependent. These results are fully reported in (Fanghella et al. 2022).

### 3.2 SEP Group Differences

The most relevant result from the ERP analysis shows significantly reduced amplitude of the (VEP‐free) SEP P100 component in ASD compared to TD in emotion but not gender task.

Specifically, results indicated enhanced somatosensory responses during emotion discrimination task in the TD compared with the ASD group, particularly in frontal and dorsal regions. This was highlighted by follow‐up analyses on significant Group × Task × Region and Group × Task × Site interactions, revealing enhanced somatosensory responses in TD compared with ASD during emotion discrimination in the frontal (two‐tailed independent‐sample *t*‐test: t(36) = 2.054, *p* = 0.047, Cohen's *d* = 0.666) and the dorsal site (two‐tailed independent‐sample *t*‐test: t(36) = 2.311, *p* = 0.027, Cohen's *d* = 0.750). Moreover, the overall activity during emotion task was enhanced in TD compared with ASD (follow‐up on the significant Group × Task interaction: main effect of Group in emotion task: F(36,1) = 6.51, *p* = 0.015, η_p_^2^ = 0.15). All these effects were not significant for gender task (all *p*‐values > 0.395, all *t* < −0.860, all Cohen's *d* < −0.279). In addition, in the TD group, follow‐up analyses showed that somatosensory responses were significantly enhanced for emotion task compared with gender task in the frontal region (two‐tailed paired‐sample *t*‐test: t(18) = 2.166, *p* = 0.044, Cohen's *d* = 0.497). In the ASD group, we found no significant differences between somatosensory responses during emotion and gender task (*p* = 0.171, *t* = −1.427. Cohen's *d* = −0.327). The full analysis of SEP components (P50, N80, P100, and N140) of this sample are reported in (Fanghella et al. 2022).

### 3.3 DCM Model Comparison

This analysis revealed that EEG data (VEP‐free SEP) were better explained by the model posing intrinsic changes in rS1 and the family modeling intrinsic changes within cortical areas.

**FIGURE 3:**
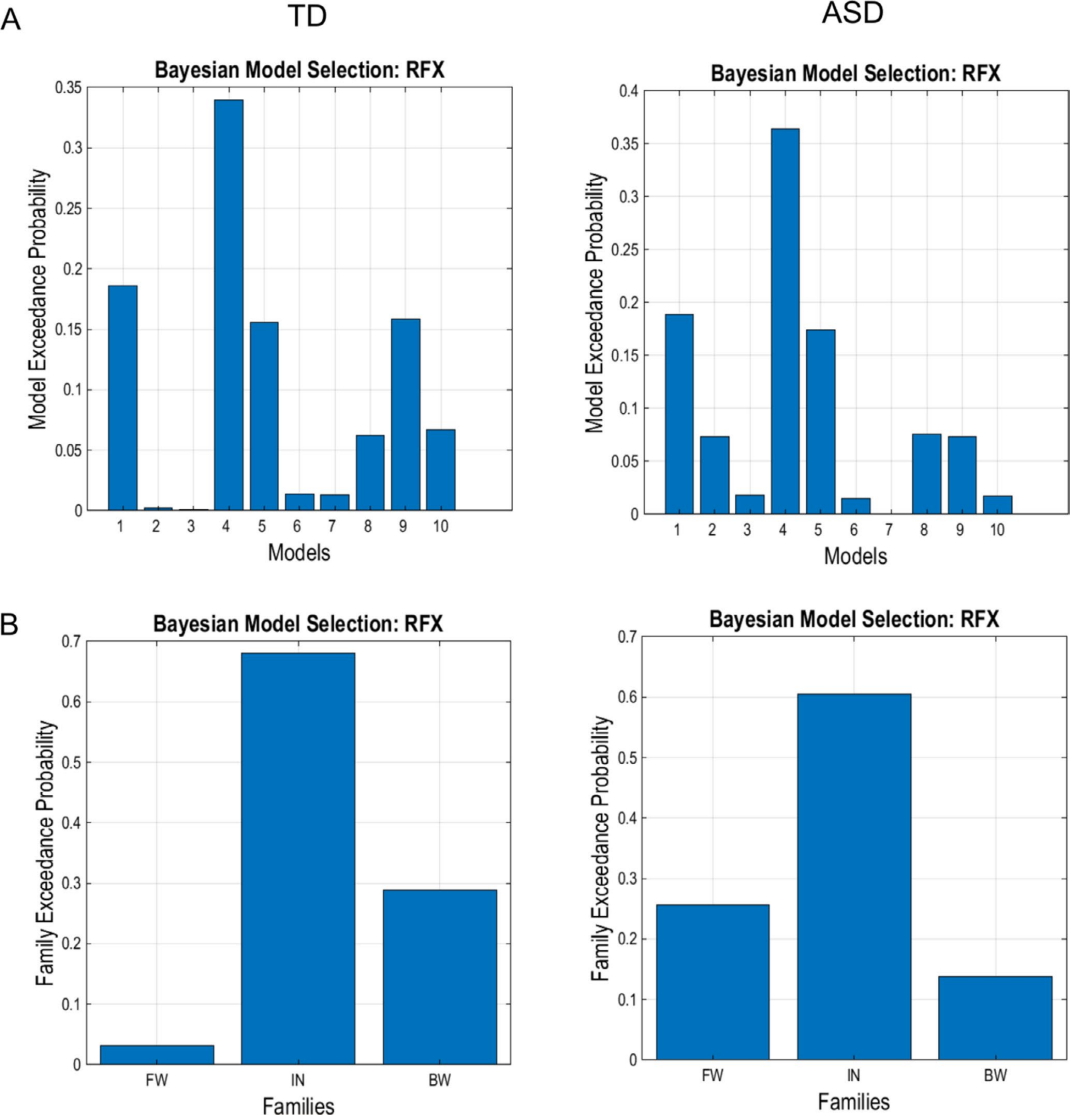
Bayesian model selection over individual data from typically developing individuals (TD; *N* = 19) and autistic individuals (ASD; *N* = 19). (A) Winning models for TD (left) and ASD (right). For both groups, the winning model is number 4: Changes in intrinsic connectivity within the right primary somatosensory cortex. (B) The winning family involves models with intrinsic task‐related modulations of brain areas in both TD (left) and ASD (right). Models: Forward (FW) family: (1) rS1 → rS2; (2) rS1 → rSMA; (3) rSMA → rIFG. Intrinsic (IN) family: (4) rS1; (5) rS2; (6) rSMA; (7) rIFG. Backward (BW) family: (8) rS2 → rS1; (9) rSMA → rS2; (10) rIFG → rSMA.

The winning model for both groups posed that task‐related differences in electrophysiological activity were caused by changes in intrinsic connectivity of rS1 (model 4: exceedance probability TD 33%, ASD 36%). The winning family was formed by four models including task‐related modulations to intrinsic connectivity of rS1, rS2, rSMA, and rIFG (intrinsic family: exceedance probability TD 68%, ASD 60%). Results from Bayesian model selection are presented in Figure 3, showing model exceedance probabilities for the 10 models and the three families.

### 3.4 DCM Effective Connectivity Parameters

This analysis showed significantly reduced task‐related intrinsic modulation of rS1 in the ASD compared to TD participants both when considering connectivity parameters from the winning model and the winning family. These parameters significantly correlated with individual alexithymia traits in the whole sample and in the autistic group.

### 3.5 Winning Model

Results from the Shapiro–Wilk normality tests on connectivity values extracted from the BMS winning model, posing task‐related intrinsic changes in rS1 (including forward and backward connections and task‐related intrinsic modulations of rS1) highlighted that connectivity values were not normally distributed (all *p*s < 0.05, all W < 0.937), except for the backward connection rSMA → rS2 (*p* = 0.069, W = 0.947). Therefore, we computed independent‐sample non‐parametric tests (*U* Mann–Whitney) for all values except for the SMA → S2 connection, where we performed an independent‐sample *t*‐test.

Independent‐sample *U* Mann–Whitney test highlighted significant group differences in task‐related modulations of intrinsic connectivity within the rS1 (*U* = 268, *p* = 0.010, two‐tailed, with a medium to large effect size, rank biserial correlation = 0.485; CI: 0.156, 0.717; TD: Mean = 0.097, SE = 0.048, ASD: Mean = −0.021, SE = 0.023) with TD showing higher modulation. No differences were found in forward or backward connectivity (all *p*s > 0.11, all rank biserial correlations < −0.307).

To further explore the association between personality traits and task‐related modulations of intrinsic connectivity within rS1, we computed non‐parametric correlations (Spearman rho (*ρ*)) between individual modulatory parameters of rS1 and personality traits (autism, alexithymia, and interoceptive awareness, measured with SRS‐2 and AQ, TAS‐20, and MAIA‐2, respectively). First, we computed correlations on the whole sample of participants, including TD and ASD individuals. Then, we tested correlations for the TD and ASD groups separately. Results highlighted significant negative correlations between individual levels of alexithymia and task‐related modulations of intrinsic connectivity in rS1 in the whole sample of participants (*n* = 38, Spearman's rho (*ρ*) = −0.377, *p* = 0.015, two‐tailed, with a medium effect size; CI from 1000 bootstraps: −0.091, −0.654) and in the ASD group (*n* = 19, Spearman's rho (*ρ*) = −0.622, *p* = 0.004, two‐tailed, with a large effect size, CI from 1000 bootstraps: −0.225, −0.868) but not in TD (all *p*s > 0.05; for full results, see Table S2). Correlations with SRS‐2, AQ, and MAIA‐2 were not significant (all *p* > 0.05; for full results, see Table S2).

### 3.6 Winning Family

Shapiro–Wilk normality tests on connectivity values extracted from BMA on the winning family, including models explaining task‐related changes as intrinsic modulations of rS1, rS2, rSMA and rIFG, revealed that all forward connections were normally distributed (rS1 → rS2, rS2 → rSMA, rSMA → rIFG, all *p*s > 0.25), backward connections were either normally distributed (rS2 → rS1, rIFG → rSMA, all *p*s > 0.27) or not normally distributed (rSMA → rS2, *p* = 0.002), and all intrinsic connections were not normally distributed (rS1, rS2, rSMA, rIFG, all *p*s < 0.001). We computed independent‐sample non‐parametric tests (*U* Mann–Whitney) for not normally distributed parameters and independent‐sample *t*‐tests for normally distributed parameters. Results replicated the previous pattern, with significant group differences in task‐related modulations of intrinsic connectivity within the rS1 (Mann–Whitney *U* = 268, *p* = 0.016, two‐tailed, with a medium effect size, rank biserial correlation = 0.452, CI: 0.114, 0.696); with TD showing higher modulation (TD: Mean = 0.030, SE = 0.014, ASD: Mean = −0.007, SE = 0.005), but no significant results for the other intrinsic connections, neither for forward or backward connectivity (all *p*s > 0.27, all rank biserial correlations < 0.213).

Results on correlations between intrinsic modulations of rS1 and personality traits of the whole sample of participants showed that task‐related intrinsic modulations of rS1 negatively correlated with autistic traits (AQ, *n* = 36, Spearman's rho (*ρ*) = −0.434, *p* = 0.008, two‐tailed, with a medium effect size, CI from 1000 bootstraps: −0.134, −0.658), alexithymia (TAS‐20, *n* = 38, *ρ* = −0.409, *p* = 0.011, two‐tailed, with a medium effect size, CI from 1000 bootstraps: −0.134, −0.636), and positively correlated with interoceptive awareness (MAIA‐2, *n* = 38, Spearman's rho (*ρ*) = 0.419, *p* = 0.009, two‐tailed, with a medium effect size, CI from 1000 bootstraps: 0.656, 0.147). The negative correlation with SRS‐2 was not significant (*n* = 33, Spearman's rho (*ρ*) = −0.311, *p* = 0.079, two‐tailed, with a medium effect size, CI from 1000 bootstraps: 0.036, −0.606). No other significant correlations were found for intrinsic modulations of rS2, rSMA, and rIFG (all *p*s > 0.05). We also found an uncorrected significant negative correlation between intrinsic modulations of rS2 and alexithymia only in the ASD group (TAS‐20, *n* = 19, Spearman's rho (*ρ*) = −0.501, *p* = 0.029, two‐tailed, with a large effect size, CI from 1000 bootstraps: −0.033, −0.811). All other correlations for ASD and TD were not significant (all *p*s > 0.05; for full results, see Table S2).

**FIGURE 4:**
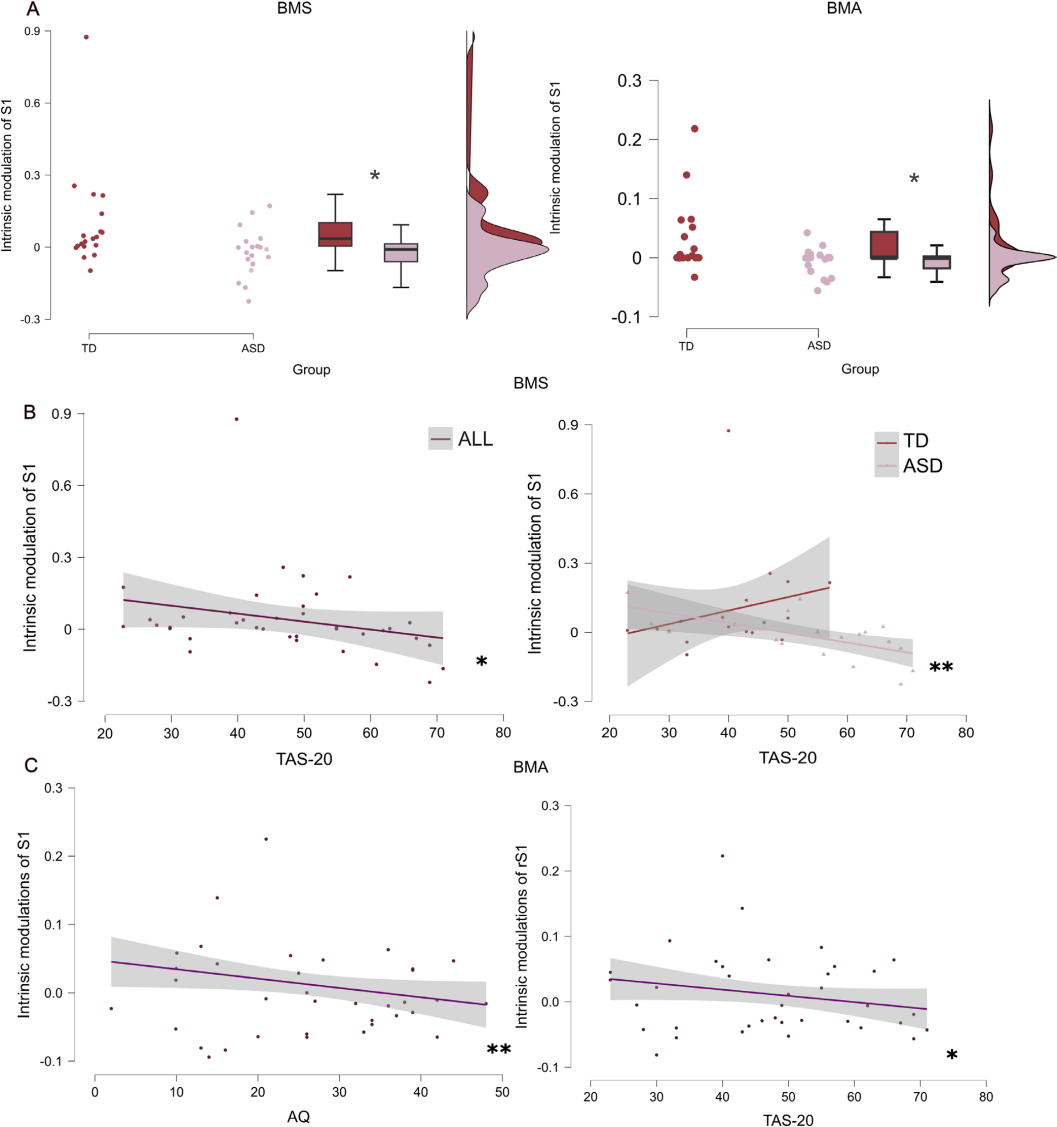
Results. (A) Raincloud plots showing significant group differences (typically developing (TD) and autistic (ASD) individuals) in task‐related modulations of intrinsic connectivity of the right primary somatosensory cortex (rS1) in Bayesian Model Selection (BMS) and Bayesian Model Averaging (BMA). (B) Correlations between alexithymia traits, measured with TAS‐20 and task‐related modulatory parameters of intrinsic connectivity of rS1 for BMS in all participants (left) and in TD and ASD separately (right). (C) Correlations between autistic traits, measured with AQ (left), and alexithymia, measured with TAS‐20 (right), and task‐related modulations of rS1 intrinsic connectivity for BMA in all participants. Task‐related increase of intrinsic connectivity in rS1 is associated with lower autistic (AQ) and alexithymia traits (TAS‐20). *p < 0.05, **p < 0.01, corrected for multiple comparisons.

Results of independent‐sample tests and correlations for BMS and BMA are depicted in Figure 4.

## 4 Discussion

This study investigated for the first time changes in effective connectivity across fronto‐parietal areas during emotion perception in two groups of ASD and TD individuals by means of DCM for EEG.

We hypothesized a network of areas lateralized in the right hemisphere, involving the rS1 and rS2, the rSMA and the rIFG, following previous models of embodied emotion (Bastiaansen et al. 2009; Goldman and Sripada 2005; Heberlein and Atkinson 2009; Hennenlotter et al. 2005; Keysers et al. 2010; Keysers and Gazzola 2009; Niedenthal 2007).

Then, we tested if task‐related group differences in early and mid‐latency SEP components (including the P50, N80, P100, and N140), co‐occurring with visual analysis of facial emotional expressions, could be better explained in terms of extrinsic (bottom‐up or top‐down) connectivity between these areas, consistently with evidence for hierarchical emotion processing (Dima et al. 2011), or in terms of changes in intrinsic connectivity within a specific area (Kiebel et al. 2007).

### 4.1 Changes in rS1 Intrinsic Connectivity Explain Differences in Emotion Perception in ASD

Our BMS results highlighted that task‐related differences (emotion versus gender discrimination) were better explained by the model posing changes in intrinsic connectivity within the rS1 both in autistic and typically developing individuals, suggesting that somatosensory processing of emotional expressions is not a by‐product of top‐down modulations from higher‐order fronto‐parietal areas. These results were confirmed by BMA, which highlighted as winning family the intrinsic modulations family, compared to forward or backward. Importantly, we compared differences in task‐related intrinsic changes within rS1 between ASD and typically developing participants. Consistently with our hypothesis, task‐related modulations of intrinsic connectivity within the rS1 were enhanced in TD compared to ASD individuals both in the winning model and in the winning family, causally explaining differences in SEP amplitudes (Fanghella et al. 2022).

Connectivity across areas included in the embodied emotion network did not differ between the two groups, except for task‐related functional changes in rS1. This contradicts previous findings suggesting altered effective connectivity in fronto‐parietal regions involved in social information processing in ASD (Cheng et al. 2015; Ecker et al. 2013; Leyhausen et al. 2024; Shih et al. 2010; Wicker et al. 2008), see (Keysers et al. 2024) for a recent review.

Our results highlight the causal role of the rS1 in processing visually presented facial emotional expressions (Arslanova et al. 2023; Fanghella et al. 2022; Sel et al. 2014, 2020) and provide evidence for hierarchical models of embodied emotion perception (Pitcher et al. 2008). This complements other accounts suggesting that the secondary somatosensory cortex (Keysers et al. 2010) or feedback and feedforward connections between fronto‐parietal areas (Isakoglou et al. 2023; Keysers et al. 2024) are crucial for typical and atypical vicarious emotional activations.

### 4.2 DCM on VEP‐Free SEP

Notably, this is the first study using DCM on VEP‐free SEPs. By subtracting visual evoked potentials, elicited by visual presentation of facial emotional stimuli, from mixed visual and somatosensory responses, this method allows to isolate somatosensory modulations from visual carryover effects (Galvez‐Pol et al. 2020). However, this subtractive method does not allow isolating somatosensory activity from modulatory effects of higher‐order areas. The current study shows that differences within the embodied emotion network between ASD and TD individuals arise from differential modulations of intrinsic activations of the rS1 and are not a byproduct of top‐down modulatory effects, as other authors have hypothesized regarding social difficulties in ASD (Hamilton 2013).

### 4.3 Task‐Related Connectivity Changes in rS1 in ASD Are Associated With Alexithymia

Importantly, our results suggest that changes in intrinsic modulatory activity of rS1 during emotion discrimination are mediated by alexithymia. In fact, we found negative correlations between the strength of participants' alexithymia traits, measured with the TAS‐20, and task‐related changes in intrinsic modulatory activity of rS1 both in the winning model (BMS) and the winning family (BMA). This indicates that higher traits in alexithymia were associated with lower levels of task‐related intrinsic changes in rS1.

Moreover, task‐related changes of rS1 within the winning family were associated with autistic traits and interoceptive awareness. These results are consistent with previous literature suggesting that alexithymia is separate from autism itself but is often associated with emotional symptoms and difficulties with interoception in ASD (Bird and Cook 2013; Garfinkel et al. 2016; Shah et al. 2016).

Some authors even argued that emotional difficulties within the autistic population are attributable to alexithymia, rather than a feature of autism per se (Cook et al. 2013). For instance, Gaigg et al. (2016) suggested that alexithymia involves a disruption in how physiological arousal modulates the subjective feelings of emotional states. However, while previous findings found a relationship between emotional empathy, levels of alexithymia and brain activity in the anterior insula (Bernhardt et al. 2014; Silani et al. 2008), no studies have yet described an association between modulations of activity in rS1, emotion processing and levels of alexithymia in the autistic population. Arslanova and colleagues (Arslanova et al. 2023) found that somatosensory activations associated with emotion processing were shaped by alexithymia in the general population.

In the current study, we observe a systematic association between levels of alexithymia and changes in somatosensory activations during emotion or gender discrimination in the ASD group and in the whole sample of participants. These results strengthen previous findings describing a crucial role of co‐occurring alexithymia in mediating difficulties in the domain of emotional processing in ASD (Bird and Cook 2013; Cook et al. 2013) and provide the first evidence of the relationship between emotion‐related functional modulations of the primary somatosensory cortex and participants' levels of alexithymia. In addition, they suggest that rS1 modulations might be associated with autistic traits and interoceptive awareness (Garfinkel et al. 2016), reflecting the complexity of emotional difficulties in ASD.

### 4.4 Limitations

A limitation of this study focused on a restricted subgroup of autistic adults, mainly male (95%), with average to above‐average intelligence and no language or learning difficulties. Although the impact on gender and IQ has not been investigated yet, it might have influenced the neural dynamics underlying somatosensory embodiment. To overcome this limitation, future research would benefit from extending these findings to different samples of autistic individuals (e.g., female, below‐average IQ, children/adolescent). Moreover, testing two groups of autistic and non‐autistic participants matched for alexithymia could be useful to disentangle autism‐related group differences from those associated with alexithymia.

Because of these limitations, we consider relevant to include constraints on generality (CoG) (Simons et al. 2017), an increasingly common practice in cognitive neuroscience research (e.g., Lopez‐Martin et al. 2026). The present findings should be interpreted considering the characteristics of the sample and the experimental context. The results are expected to generalize primarily to autistic adults with average to above‐average intelligence, preserved language abilities, and similar demographic characteristics to those tested here. Given that the autistic sample was predominantly male, the extent to which these findings generalize to female or gender‐diverse autistic individuals remains unclear. Moreover, the effects were observed using a specific EEG paradigm involving visual emotion and gender discrimination tasks combined with task‐irrelevant tactile stimulation; thus, generalization to other forms of emotion processing, sensory modalities, or experimental designs should be made cautiously. Finally, because the study focused on a restricted set of cortical regions within a predefined embodied emotion network, the present conclusions may not extend to broader neural systems involved in emotion processing beyond those examined here.

## 5 Conclusions

Our results provide evidence that decreased somatosensory evoked potentials associated with differences in emotion processing in ASD are causally explained by reduced functional modulation of intrinsic activity in rS1, rather than bottom‐up or top‐down modulations between sensorimotor and frontal areas. Moreover, they suggest that emotion‐related activations in rS1 may be mediated by alexithymia, interoceptive awareness, and autistic traits. Future research will clarify how other areas crucial for emotion processing, such as the amygdala or the anterior insula, interact with sensorimotor areas to contribute to differences in emotion perception in ASD. Moreover, it will expand our understanding of the link between somatosensory processing of emotions, autistic traits, alexithymia, and interoception in individuals with and without ASD (Garfinkel et al. 2016; Palser et al. 2020, 2021; Shah et al. 2016).

## Author Contributions

Conceptualization: Martina Fanghella, Danai Dima, Dimitrios Pinotsis, Sebastian B. Gaigg, Beatriz Calvo‐Merino, and Bettina Forster. Methodology: Martina Fanghella, Danai Dima, Dimitrios Pinotsis, Beatriz Calvo‐Merino, and Bettina Forster. Software: Martina Fanghella, Danai Dima, and Dimitrios Pinotsis. Formal analysis: Martina Fanghella, Danai Dima, Dimitrios Pinotsis, and Bettina Forster. Investigation: Martina Fanghella, Sebastian B. Gaigg, Beatriz Calvo‐Merino, and Bettina Forster. Writing – original draft: Martina Fanghella, Danai Dima, and Bettina Forster. Writing – review and editing: Martina Fanghella, Danai Dima, Dimitrios Pinotsis, Sebastian B. Gaigg, Beatriz Calvo‐Merino, and Bettina Forster. Visualization: Martina Fanghella. Supervision: Bettina Forster. All authors commented on and approved the manuscript.

## Funding

D.P. was supported by the Medical Research Council (Grant number MR/W011751/1). M.F. was funded by a PhD scholarship from the joint PhD program in Psychology and Social Neuroscience, City St George's, University of London and La Sapienza, University of Rome, and from the Department of Philosophy ‘Piero Martinetti’ of the University of Milan, with the Project “Departments of Excellence 2018‐2022”, awarded by the Italian Ministry of University and Research (MUR).

## Ethics Statement

All participants provided written informed consent, and the Ethical Committee of City, University of London approved the original study (PSYETH (S/F) 17/18 105).

## Consent

The authors have nothing to report.

## Conflicts of Interest

The authors declare no conflicts of interest.

## Supporting information


**Table S1:** Demographics and questionnaire scores for ASD and TD participants.
**Table S2:** Results from correlations between task‐related changes in intrinsic activity of right primary somatosensory cortex (rS1) and individual scores in personality questionnaires measuring autistic traits (SRS‐2 and AQ), alexithymia (TAS‐20) and interoceptive awareness (MAIA‐2). *ρ*: Spearman's rho. *p < 0.05; **p < 0.01.

## Data Availability

The data that support the findings of this study are available on request from the corresponding author. The data are not publicly available due to privacy or ethical restrictions.
