# Supplementary material for: Changes in Intrinsic Activity of the Primary Somatosensory Cortex Causally Explain Differences in Emotion Perception in Autism
Source: Autism Res. 2026 Feb 13;19(4):e70197. doi: 10.1002/aur.70197 (PMC13087861; doi:10.1002/aur.70197)
Supplement: Supplementary file 1 — Table S1: Demographics and questionnaire scores for ASD and TD participants. Table S2: Results from correlations between task‐related changes in intrinsic activity of right primary somatosensory cortex (rS1) and individual scores in personality questionnaires measuring autistic traits (SRS‐2 and AQ), alexithymia (TAS‐20) and interoceptive awareness (MAIA‐2). ρ: Spearman's rho. *p < 0.05; **p < 0.01. [file AUR-19-0-s001.docx]

**Supplementary Tables**

*Table S1.* Demographics and questionnaire scores for ASD and TD participants.

|  | TD | ASD | Results |
| --- | --- | --- | --- |
| Age | 40.84 ± 12.24 | 40.47 ± 8.86 | t(36) = 0.11, p = 0.92 |
| VIQ | 113.58 ± 17.80 | 108.56 ± 15.38 | t(35) = 0.92, p = 0.37 |
| PIQ | 117.42 ± 13.98 | 111.17 ± 14.75 | t(35) = 1.32, p = 0.194 |
| SRS-2 | 49.29 ± 5.91 | 69.12 ± 11.37 | t(32) = 6.39, p = 0.000** |
| AQ | 17.61 ± 8.79 | 34.89 ± 7.76 | t(34) = 6.25, p = 0.000** |
| TAS-20 | 40.42 ± 8.76 | 54.33 ± 14.19 | t(36) = 3.63, p = 0.000** |
| MAIA-2 | 3.15 ± 0.68 | 2.65 ± 0.81 | t(36) = 3.44, p = 0.048* |

*Table S2.* Results from correlations between task-related changes in intrinsic activity of right primary somatosensory cortex (rS1) and individual scores in personality questionnaires measuring autistic traits (SRS-2 and AQ), alexithymia (TAS-20) and interoceptive awareness (MAIA-2). ρ: Spearman’s rho. *p <.05; **p <.01.

|  | SRS-2 | AQ | TAS-20 | MAIA-2 |
| --- | --- | --- | --- | --- |
| All participants | ρ = -.249  p = .162  n = 33 | ρ = -.310  p = .066  n = 36 | ρ = -.392*  p = .015  n = 38 | ρ = .255  p = .122  n = 38 |
| TD participants | ρ = .366  p = .148  n = 17 | ρ = .292  p = .240  n = 18 | ρ = .395  p = .095  n = 19 | ρ = .207  p = .393  n = 19 |
| ASD participants | ρ = .240  p = .370  n = 16 | ρ = -.041  p = .870  n = 18 | ρ = -.622**  p = .004  n = 19 | ρ = .047  p = .874  n = 19 |
